# Mitochondrial dynamics in cisplatin resistance: molecular mechanisms and therapeutic targeting

**DOI:** 10.3389/fonc.2025.1736487

**Published:** 2026-01-07

**Authors:** Wei Huang, Wei Sun, Zhiyuan Yang, Yiwen Li, Ziliang Wang

**Affiliations:** 1 Science and Technology Innovation Center, Shanghai Municipal Hospital of Traditional Chinese Medicine, Shanghai University of Traditional Chinese Medicine, Shanghai, China; 2 Department of Gynecology, Shanghai Municipal Hospital of Traditional Chinese Medicine, Shanghai University of Traditional Chinese Medicine, Shanghai, China

**Keywords:** cisplatin resistance, metabolic reprogramming, mitochondrial dynamics, mitophagy, targeted therapy, immune microenvironment

## Abstract

Cisplatin remains a cornerstone of chemotherapy for numerous cancers, despite the persistent challenges of toxicity and the development of drug resistance. Therefore, a deeper understanding of the mechanisms behind cisplatin resistance and the development of strategies to counter it are of critical importance. This review systematically examines the pivotal role of mitochondrial dynamics in cisplatin resistance and discusses emerging therapeutic strategies that target these processes. Mitochondrial dynamics regulate the structure and function of the mitochondrial network through a balance of fusion and fission. Dysregulation of this process directly contributes to cisplatin resistance by maintaining cellular energy homeostasis, inhibiting apoptosis, and enhancing oxidative phosphorylation (OXPHOS). Furthermore, mitophagy, metabolic reprogramming, and the tumor immune microenvironment converge on mitochondrial dynamics to drive the acquisition of drug resistance. Consequently, targeting mitochondrial dynamics presents a promising approach to overcome cisplatin resistance. Potential strategies include restoring the balance of fusion and fission, intervening in mitophagy, disrupting OXPHOS metabolism, and developing mitochondrial-targeted nanodrug delivery systems. However, despite this promising outlook, significant challenges remain, including the heterogeneity of resistance mechanisms, a lack of reliable biomarkers, and the need for selective targeting to minimize off-target effects.

## 1 Introduction

Mitochondrial dynamics refer to the dynamic changes in the morphology, distribution, and function of the mitochondrial network. This process is centrally governed by the balanced regulation of fission and fusion. In essence, it encompasses the temporal evolution of the network’s morphological features and connectivity, achieved primarily through the continual structural remodeling driven by these two opposing mechanisms (1). Mitochondrial fission is the process by which elongated tubular mitochondria are divided into separate organelles. This process is primarily mediated by proteins such as MFF and Drp1 (2). The Drp1 protein facilitates mitochondrial fission by forming helical structures that constrict and sever the mitochondrial outer membrane, a process powered by GTP hydrolysis (3). In contrast, mitochondrial fusion is the process by which separate mitochondria merge to form elongated, branched, and interconnected tubular networks. This event is orchestrated by the coordinated actions of the outer membrane proteins Mfn1 and Mfn2, and the inner membrane protein OPA1 (4, 5). The overall morphology of the mitochondrial network is determined by the dynamic balance between fission and fusion. An excess of fission results in a fragmented network, while a predominance of fusion promotes a highly interconnected morphology (6). Mitochondrial fission facilitates network fragmentation by isolating damaged mitochondrial components for removal via mitophagy (7), This adaptive shift promotes glycolysis in cancer cells (8). Mitochondrial fusion enables functional complementation by diluting damaged components, such as mutated mitochondrial DNA (mtDNA), thereby helping to maintain overall mitochondrial functional integrity (4). Furthermore, the formation of an interconnected network through fusion enhances the efficiency of cellular oxidative phosphorylation (6). Furthermore, the formation of an interconnected network through fusion enhances the efficiency of cellular oxidative phosphorylation (6). In summary, mitochondrial dynamics serve as a crucial mechanism for maintaining cellular homeostasis. The precisely regulated balance between fission and fusion enables cells to remodel organelle morphology, control quality, and adapt energy production to meet metabolic demands. Dysregulation of this process is strongly implicated in the pathogenesis of diverse diseases, including neurodegenerative disorders, metabolic diseases, and cancer. Consequently, developing targeted therapeutic interventions against key regulators of mitochondrial dynamics, such as the proteins Drp1 and Mfn2, has become a major focus of current research (9, 10).

Cisplatin is a chemotherapeutic agent extensively used in the treatment of various solid tumors. Nevertheless, its clinical efficacy is substantially limited by the development of drug resistance and tumor recurrence (11–13). Nevertheless, cisplatin-based combination chemotherapy can achieve curative outcomes in highly sensitive malignancies, such as testicular cancer. Research indicates that clinical response rates to cisplatin vary substantially across different cancer types. For instance, response rates to cisplatin are as low as 20% in non-small cell lung cancer (NSCLC) (14), In contrast, response rates exceed 90% in testicular cancer (15). The mechanisms underlying cisplatin resistance are highly heterogeneous. Firstly, the overexpression of ABC transporter proteins—such as ABCB1/P-gp, ABCC1, and ABCG2—can mediate cisplatin efflux from cells, thereby contributing to multidrug resistance (MDR). This mechanism is particularly enriched in cancer stem cells (CSCs) (16, 17). In mesothelioma stem cells, activation of ABCB5 has been identified as a key mediator of cisplatin resistance (18). Furthermore, dysregulation of DNA repair mechanisms represents another major pathway. Tumor cells can counteract cisplatin-induced DNA damage by enhancing their repair capacity. For instance, in nasopharyngeal carcinoma, overexpression of FOX family transcription factors such as FoxM1 and FOXQ1 promotes this protective response (16). Additionally, inhibition of apoptosis significantly contributes to cisplatin resistance. This can occur through overexpression of anti-apoptotic proteins such as Bcl-2 and Bcl-xL, which suppress cisplatin-induced cell death. Alternatively, loss of pro-apoptotic function, as seen with dysfunctional mutant p53 in gastric cancer, also confers treatment resistance (19);In nasopharyngeal carcinoma, TBL1XR1 contributes to resistance by activating the NF-κB signaling pathway, which subsequently inhibits apoptosis (16). Epigenetic regulation also plays a significant role; non-coding RNAs (ncRNAs), such as lncRNA HOTAIR and circCCND1, promote resistance through multiple mechanisms including the modulation of reactive oxygen species (ROS) levels, the enhancement of cancer stem cell properties, and the facilitation of DNA repair processes (20). Dysregulation of miRNAs, such as the downregulation of miR-149-3p, can enhance cisplatin resistance in lung cancer by targeting genes like TMPRSS4 (21, 22). Within the tumor microenvironment (TME), the epithelial-mesenchymal transition (EMT) process—for instance, Snail-induced reduction of E-cadherin—can enhance P-gp function and promote drug efflux (17). Autocrine signaling loops, such as the Wnt/IL-1β/IL-8 pathway, can induce ABCB5 expression in mesothelioma stem cells (18). Regarding metabolic and stress adaptations, endoplasmic reticulum stress (ERS) and the unfolded protein response (UPR) have been linked to platinum resistance in high-grade serous ovarian carcinoma (HGSOC) (23). Mitophagy exhibits a dual regulatory role in drug resistance in liver cancer: pro-survival mitophagy enhances chemoresistance, whereas pro-death mitophagy suppresses tumor growth (24). To address cisplatin resistance, current strategies primarily involve combination therapies, including targeted agents, epigenetic modulators, and immunotherapy. For example, in drug combinations, PARP inhibitors are used synergistically with cisplatin to exacerbate DNA damage in tumor cells. However, such approaches still face challenges related to safety, such as off-target effects and the risk of viral reactivation (25). In epigenetic interventions, exosome-mediated delivery of siRNA (e.g., targeting the CPT1A gene) has shown potential to reverse cisplatin resistance in gastric cancer, though optimization of delivery efficiency and targeting specificity remains necessary (26). In immunotherapy, immune checkpoint inhibitors (ICIs) demonstrate efficacy in certain tumors; however, their utility is complicated by complex resistance mechanisms, such as T-cell dysfunction (16). In summary, cisplatin resistance remains a formidable obstacle in cancer therapy, arising from highly complex and multifactorial biological processes. Current research efforts are primarily focused on developing combination strategies involving targeted therapy, epigenetic modulation, and immunotherapy. However, significant challenges persist, including the inherent complexity of resistance mechanisms, a scarcity of reliable biomarkers, and difficulties in clinical translation.

Mitochondria play multifaceted roles in cellular physiology, functioning not only as the primary site of energy metabolism but also as key regulators of drug tolerance. Under hypoxic conditions, reduced oxidative damage to mtDNA confers enhanced resistance to cisplatin in tumor cells (27). Furthermore, the upregulation of mitochondrial apurinic/apyrimidinic endonuclease (mtAPE1) in osteosarcoma-resistant cells contributes to chemoresistance by clearing oxidatively damaged mtDNA and suppressing ROS production, thereby protecting cells from cisplatin-induced cytotoxicity (28). Collectively, these findings highlight an essential link between mtDNA damage repair mechanisms and cisplatin resistance in cancer cells. Therefore, mitochondrial-targeted strategies show significant potential for overcoming cisplatin resistance. For example, drug delivery systems designed to target mitochondria—such as those using nanoparticles or liposomes to transport cisplatin directly to these organelles—can bypass the nuclear DNA repair mechanisms that often confer drug resistance (29). In summary, mitochondria contribute to cisplatin resistance through a spectrum of mechanisms that exhibit distinct tumor-type specificity and microenvironmental dependencies. Consequently, mitochondrial-targeted interventions represent a promising strategic avenue for effectively reversing cisplatin resistance.

## 2 The mechanistic role of key mitochondrial dynamics regulators in cisplatin resistance

### 2.1 Imbalance between fusion and fission

A marked correlation exists between mitochondrial dynamics and cisplatin resistance. In cisplatin-resistant cells, the upregulation of key fusion proteins Mfn1, Mfn2, and OPA1 promotes mitochondrial elongation. This morphological alteration helps maintain cellular energy homeostasis and effectively imparts resistance to apoptosis (30, 31). Investigations in non-small cell lung cancer have revealed that mitochondrial fusion, mediated by the p32/OPA1 axis, directly promotes cisplatin resistance (32). In lung adenocarcinoma, elevated OPA1 expression similarly enhances cisplatin resistance by inactivating the caspase-dependent apoptotic pathway (33). In tongue squamous cell carcinoma (TSCC), mitochondrial fission factor (MFF) mediates both mitochondrial fission and apoptosis following cisplatin treatment. During this process, miR-593-5p expression is downregulated. Further investigation revealed that miR-593-5p targets the 3’ untranslated region (3’UTR) of MFF mRNA to suppress its translation, consequently attenuating both mitochondrial fission and cisplatin sensitivity (34). In cholangiocarcinoma cells, cisplatin-induced INF2 protein is degraded through both SEC62-mediated reticulophagy and the ubiquitin-proteasome system. This degradation impairs mitochondrial fission, resulting in a hyperfused mitochondrial network. This adaptive response has been identified as a key mechanism by which cholangiocarcinoma (CCA) cells resist cisplatin-induced apoptosis (35). Furthermore, Drp1 is a conserved mechanoenzyme that catalyzes mitochondrial constriction and fission through GTP hydrolysis, serving as a master regulator of mitochondrial division (36, 37). Studies demonstrate that cisplatin-resistant ovarian cancer cells exhibit significantly lower DRP1 expression levels and more pronounced mitochondrial fusion compared to their cisplatin-sensitive counterparts. This shift in mitochondrial dynamics confers increased resistance to cisplatin-induced apoptosis (38). Collectively, these findings indicate that mitochondrial fusion is a significant contributor to cisplatin resistance in cancer cells. However, other studies have reported an upregulation of the MFF in cisplatin-resistant liver cancer cells. In this context, MFF enhances chemoresistance by activating Drp1 to promote mitochondrial fission, suggesting that the mechanisms of resistance may be cell type-specific (39). Research further indicates that extracellular matrix (ECM) stiffness influences mitochondrial ROS production and NRF2 activation through the regulation of DRP1. Softer ECM promotes perimitochondrial F-actin assembly, which enhances DRP1-mediated mitochondrial fission. This fission process is coupled with increased mtROS generation, leading to NRF2 pathway activation and subsequent augmentation of cellular antioxidant defense systems. Consequently, soft ECM conditions activate both NRF2 and DRP1 signaling, enabling cancer cells to develop resistance to ROS-dependent chemotherapeutic agents, including cisplatin and arsenic trioxide (40). Therefore, mitochondrial fusion and fission processes exhibit a dual role in regulating cisplatin resistance. Nevertheless, it is evident that an imbalance between mitochondrial fusion and fission is directly linked to the development of cisplatin resistance (**Table 1**).

**Table 1:** Summary of key mitochondrial dynamics proteins and their functions in cisplatin resistance.

Target	Alteration in function	Associated cancer types	Drug resistance mechanisms	Intervention strategies	References
DRP1	Downregulation	Ovarian cancer	Mitochondrial fission inhibition→Enhanced mitochondrial fusion→Apoptosis resistance	DRP1 overexpression or treatment with a DRP1 activator	(38)
	Increased phosphorylation of DRP1 at Ser616	Breast cancer	Increased mitochondrial fission→mtROS production→Activation of the NRF2-mediated antioxidant response	Drpitor1a	(40)
MFF	Downregulation	Tongue squamous cell carcinoma (TSCC)	Mitochondrial fission inhibition→Inhibition of the cisplatin-induced apoptosis pathway	Inhibition of either miR-593-5p or BRCA1	(34)
	Upregulation	Hepatocellular carcinoma	Recruitment of Drp1 to mitochondria → Excessive mitochondrial fission → Confers cisplatin resistance in HCC	MFF knockdown or treatment with an MFF inhibitor	(39)
OPA1	Upregulation	Non-small cell lung cancer (NSCLC)	Enhanced mitochondrial fusion→Increased ATP production→apoptosis resistance.	Suppression of the p32/OPA1 signaling axis	(32)
		Lung adenocarcinoma	Maintenance of mitochondrial network architecture→inhibit cytochrome c release→Inhibition of the apoptosis pathway	OPA1 knockdown or inhibition OPA1 knockdown or treatment with an OPA1 inhibitor	(33)
Mfn1/2	Upregulation	Ovarian cancer, Neuroblastoma, Leukemia	Enhanced mitochondrial fusion→Protection of mitochondrial network integrity→apoptosis resistance.	Mfn1/2 knockdown or treatment with a mitofusin inhibitor	(30, 31)
INF2	Cisplatin-induced protein degradation	Cholangiocarcinoma	Mitotic Spindle Disruption→Highly interconnected mitochondrial network	Inhibition of the Ubiquitin-Proteasome Pathway (UPP)	(35)

### 2.2 The Janus-faced nature of mitophagy

The process of mitophagy plays a crucial role in regulating mitochondrial quality control and thereby helping to maintain normal cellular physiological functions (41–43). As two essential components of a unified mitochondrial quality control system, mitophagy and mitochondrial dynamics work in concert to preserve the integrity of the mitochondrial network and maintain cellular energy homeostasis (44). Mitochondrial dynamics and mitophagy collaboratively establish a quality surveillance network through the fission-fusion cycle and functional interactions among key proteins, including Drp1, Mfns, and the PINK1/Parkin pathway. Mitochondrial fission facilitates the isolation and subsequent autophagic removal of damaged organelles, whereas fusion enables content mixing to repair mild damage and prevent excessive mitochondrial turnover. Disruption of the fission-fusion equilibrium induces mitochondrial dysfunction, which contributes to cancer pathogenesis and is strongly associated with the development of cisplatin resistance (45–47). Elucidating the molecular mechanisms underlying mitophagy in cisplatin resistance is therefore crucial for understanding mitochondrial dynamics.

**Figure 1:**
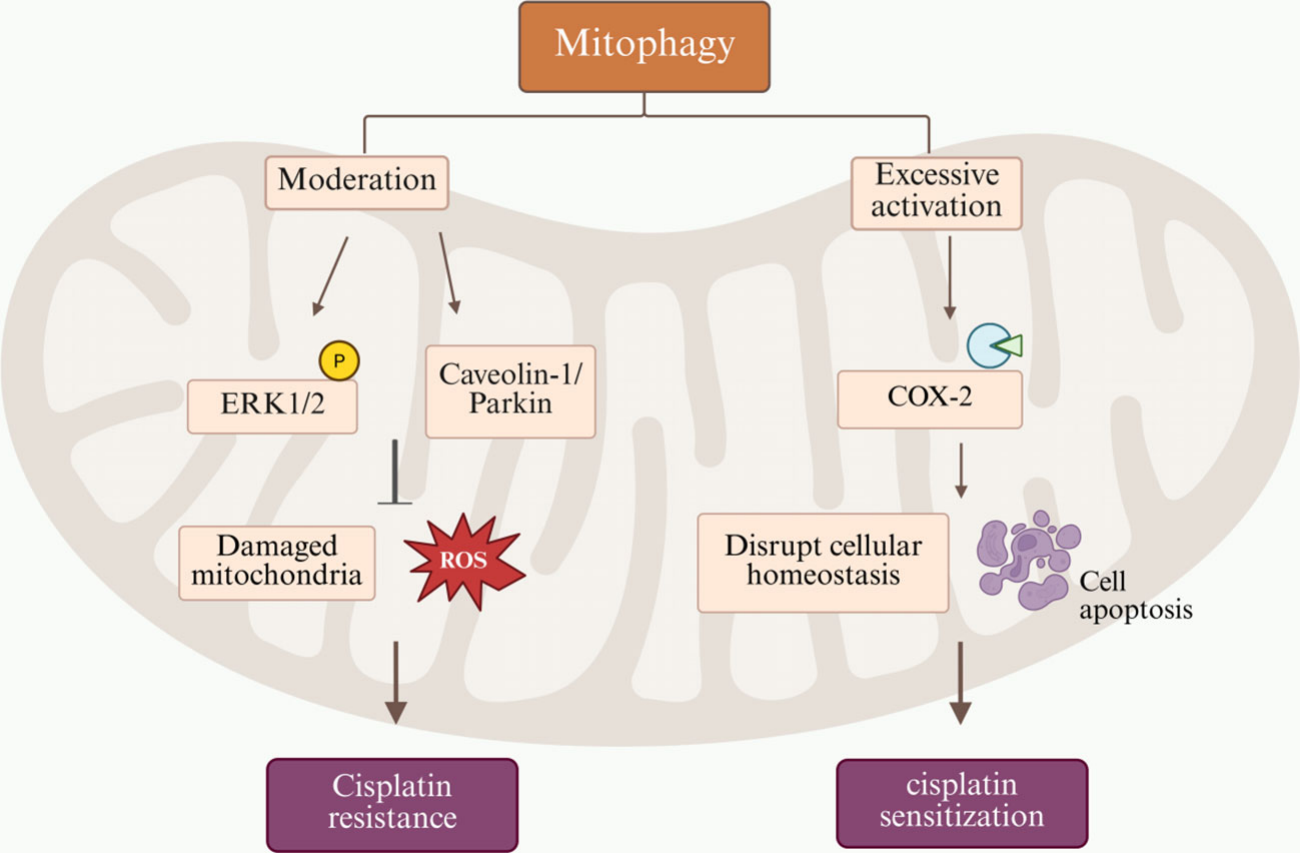
The dual role and underlying mechanisms of mitophagy in cisplatin resistance.

Mitophagy also exhibits a dual role in mitochondrial-mediated cisplatin resistance, with its effects being highly context-dependent based on factors such as cell type, physiological state, and regulatory intensity. On one hand, in cisplatin-treated malignant cells, the mitophagy mechanism promotes cell survival by clearing damaged mitochondria. This process reduces the accumulation of ROS and alleviates mitochondrial dysfunction, thereby helping cancer cells evade apoptosis. In NSCLC cells, for example, Caveolin-1/Parkin-mediated mitophagy enhances cisplatin resistance. Conversely, inhibiting mitophagy restores drug sensitivity and promotes apoptosis (48). Similarly, in ovarian cancer cells, cisplatin treatment activates the ERK signaling pathway to induce mitophagy as a compensatory mechanism to counteract its toxicity. Pharmacological inhibition of this autophagic response consequently enhances the cytotoxic effects of cisplatin (49). Conversely, excessive activation of mitophagy may lead to the unchecked removal of functional mitochondria, subsequently triggering metabolic dysregulation and energy exhaustion that ultimately induce cell death. This is supported by studies showing that hyperactive mitophagy disrupts cellular metabolic homeostasis, thereby promoting cell death (50). In hepatocellular carcinoma cells, ketoconazole promotes cisplatin-induced apoptosis by downregulating COX-2 to induce mitophagy (51). Collectively, these findings substantiate the dual role of mitophagy in the development of cisplatin resistance. At moderate levels, mitophagy clears damaged mitochondria to maintain cellular viability, thereby promoting resistance. Conversely, excessive mitophagic activity can disrupt cellular homeostasis and paradoxically enhance the cytotoxic efficacy of cisplatin (**Figure 1**).

### 2.3 Mitochondrial dynamics and cancer stemness

CSC possess intrinsic resistance mechanisms that enable them to evade cisplatin-induced cytotoxicity through pathways such as ROS scavenging and activation of anti-apoptotic signaling. This phenomenon has been observed in multiple cancer types, including ovarian cancer, mesothelioma, and nasopharyngeal carcinoma (18, 52, 53). However, mitochondrial morphological changes show a significant correlation with the maintenance of cancer stemness. In oral squamous cell carcinoma (OSCC), inhibition of the fission protein DRP1 induces mitochondrial elongation. This morphological change increases α-ketoglutarate (α-KG) levels, which promotes histone H3K27me3 demethylation. Consequently, this epigenetic modification downregulates the expression of stemness-related genes (e.g., SNAI2) and EMT programs, ultimately attenuating CSC properties (54). Research in brain tumor-initiating cells (BTICs) has demonstrated that hyperactivation of DRP1 induces mitochondrial fragmentation. This fragmentation is closely associated with enhanced self-renewal capacity, drug resistance, and invasiveness in CSCs. For instance, in lung CSCs, FIS1-mediated fission promotes mitophagy, thereby helping to maintain stemness properties (55, 56). These findings reveal that the effects of mitochondrial fusion and fission on cancer stemness exhibit context-dependent heterogeneity across tumor types. They further confirm the bidirectional regulatory role of mitochondrial dynamics in mediating cisplatin resistance.

Mitochondrial metabolism plays a pivotal role in maintaining the stemness properties of tumor cells. CSCs predominantly rely on mitochondrial OXPHOS rather than glycolysis. In CSCs derived from ovarian cancer, small cell lung cancer, and glioma, key enzymes involved in OXPHOS and fatty acid oxidation (FAO) are upregulated, providing essential metabolic support that meets their high energy demands and enhances drug resistance (57, 58). This underscores the essential role of mitochondrial metabolism in providing the energy required to maintain tumor cell stemness. However, elongated mitochondrial morphology promotes glutamine metabolism—mediated by the ASCT2 transporter—to fuel the tricarboxylic acid (TCA) cycle, while simultaneously suppressing the expression of stemness-related genes through epigenetic regulatory mechanisms (54). These findings reveal the mechanistic role of mitochondrial dynamics in regulating metabolic processes to maintain cancer stemness. Furthermore, mitochondrial retrograde signaling—a process studied in epigenetics and signal transduction—can trigger cell reprogramming resembling EMT, thereby enhancing cellular migration and invasion capabilities (59). Furthermore, activation of DRP1 during mitochondrial fission regulates stem cell differentiation fate by modulating the FOXO/Notch signaling pathway (60).

Mitochondrial dynamics play a central role in apoptosis resistance; a mechanism closely linked to the therapeutic sensitivity of cancer stem cells. According to relevant studies, the regulation of mitochondrial dynamics significantly influences the therapeutic response of cancer stem cells. Inhibition of DRP1 expression induces mitochondrial elongation, thereby enhancing the sensitivity of OSCC to the ferroptosis-inducing agent erastin (54). Therefore, modulating mitochondrial dynamics can influence the sensitivity of cancer stem cells to ferroptosis inducers, ultimately affecting therapeutic efficacy. Moreover, the fused mitochondrial state also influences cell death resistance in cancer stem cells. Overexpression of MFN2 promotes mitochondrial fusion, thereby conferring resistance to apoptosis, whereas mitochondrial fission facilitates the apoptotic process (61, 62). However, during chemotherapy, mitochondrial fragmentation may paradoxically enhance cell death resistance in certain cancer cells. This paradox highlights the context-dependent dual role of mitochondrial dynamics regulation in cancer therapy. Furthermore, CSCs can dynamically modulate mitochondrial morphology to develop resistance against OXPHOS inhibitors. For example, in acute myeloid leukemia, cancer stem cells can acquire resistance to OXPHOS inhibitors through exogenous mitochondrial transfer. This adaptive mechanism demonstrates how cancer stem cells dynamically modulate both mitochondrial structure and function to counteract therapeutic stress, thereby maintaining their survival and proliferative capacity (63).

## 3 Drug resistance mechanisms through metabolic-dynamic interplay

### 3.1 The interplay between mitochondrial dynamics and metabolism

**Figure 2:**
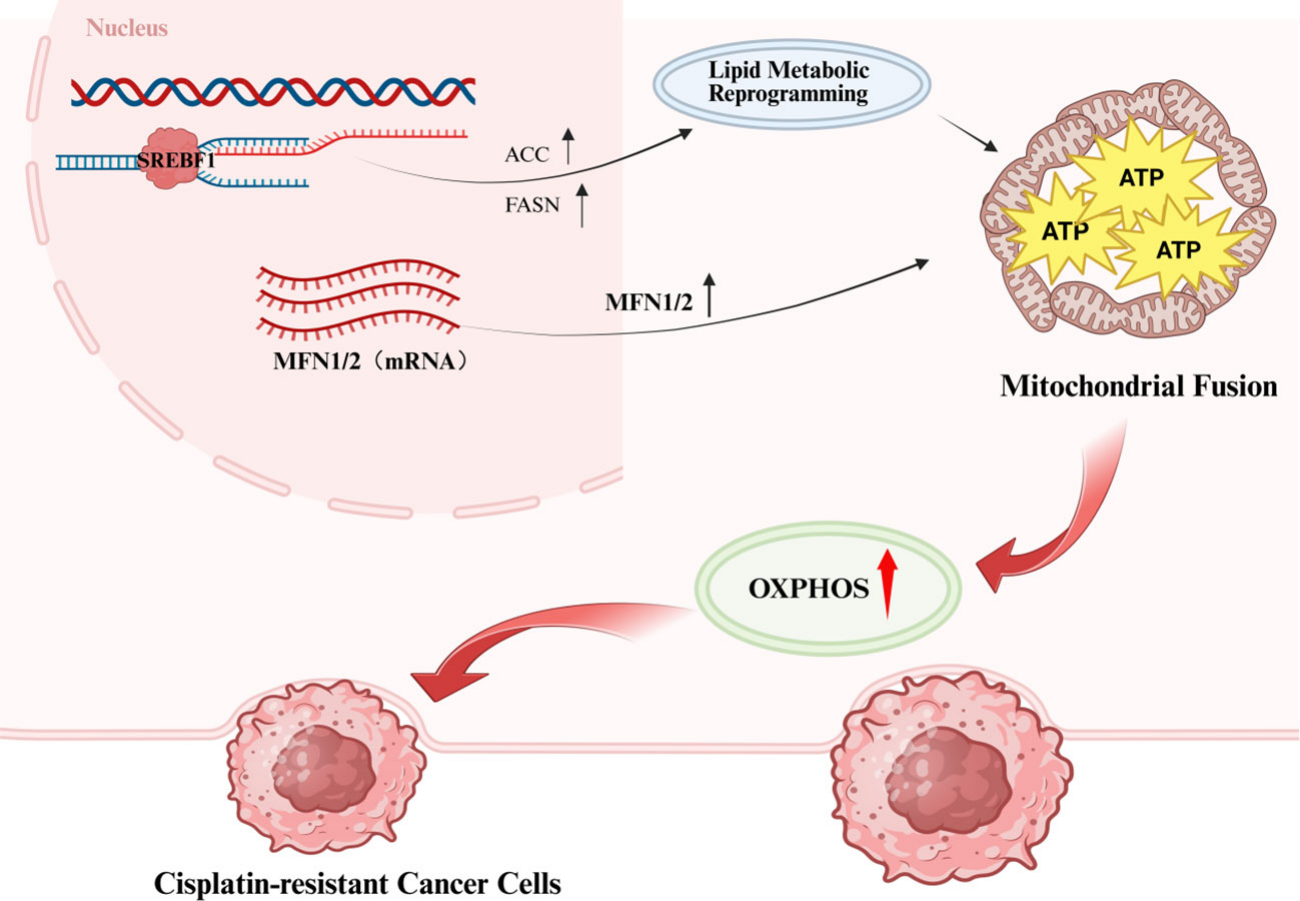
Cisplatin resistance mediated by the interplay between mitochondrial dynamics and metabolism.

Mitochondrial dynamics are intimately and inextricably linked to tumor cell metabolic processes, directly regulating cellular energy metabolism. Studies demonstrate that mitochondrial fusion enhances OXPHOS efficiency, with fused mitochondria exhibiting superior energy production capacity. Conversely, excessive mitochondrial fission can induce fragmentation of metabolic processes, ultimately disrupting normal cellular function (64–67). Cellular metabolic status can conversely regulate mitochondrial dynamics. High-glucose or high-lipid environments promote mitochondrial fission, leading to increased production of ROS. This process may ultimately disrupt cellular metabolic homeostasis (68, 69). Furthermore, GLUT4 and PGC-1α—a transcriptional co-regulator of mitochondrial genes—coordinate cellular metabolic adaptation by modulating the activity of dynamics proteins such as OPA1 (70, 71). The interactions between these molecules and proteins collectively form an intricate interrelationship between mitochondrial dynamics and tumor cell metabolism, thus holding critical scientific importance for deepening our understanding of tumorigenesis and cancer progression. In drug-resistant cancer cells, mitochondrial fusion is markedly enhanced, a process primarily underpinned by upregulated expression of mitofusin 1 and 2 (Mfn1/2). This fusion process facilitates the formation of a highly interconnected mitochondrial network, which markedly enhances cellular OXPHOS capacity. This improved bioenergetic competence enables cells to more effectively respond to environmental stressors and meet internal metabolic demands (31). Furthermore, perturbations in lipid metabolism—such as enhanced fatty acid oxidation—may further promote mitochondrial fusion through the regulatory activity of key transcription factors including sterol regulatory element-binding factor 1 (SREBF1). This adaptive response helps safeguard stable energy supply under adverse environmental conditions (72, 73). This intricate interplay between metabolic and dynamical regulatory mechanisms provides a crucial biological foundation for tumor cell survival and drug resistance (**Figure 2**).

### 3.2 The metabolic-dynamic axis mediates drug resistance

**Figure 3:**
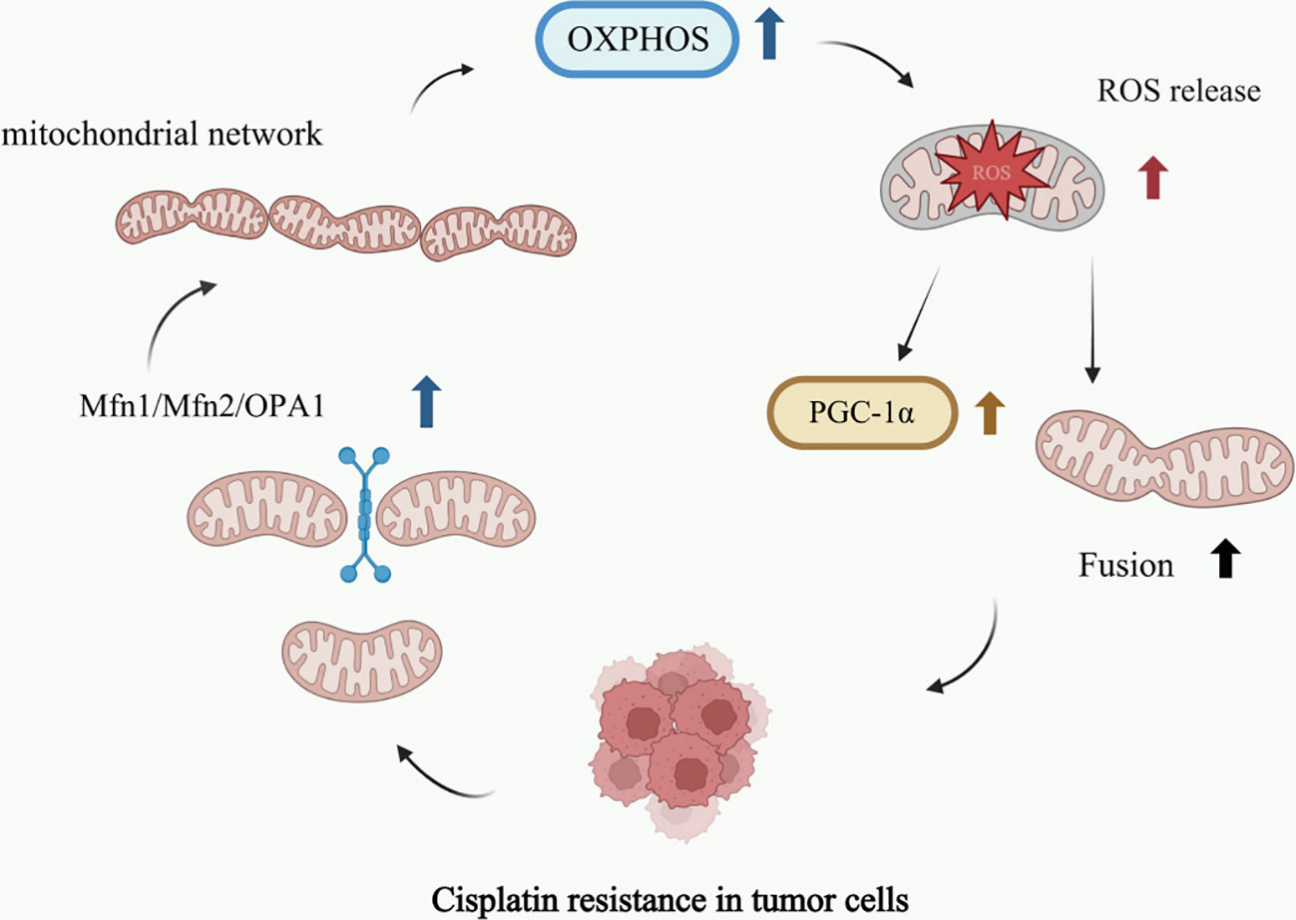
Mitochondrial dynamics affects cell metabolism to form a drug-resistant cycle.

In cancer cells, metabolic reprogramming not only alters the ways in which cells acquire and utilize energy but also profoundly impacts mitochondrial dynamics (74, 75). In cisplatin-resistant tumor cells, metabolic reprogramming occurs in response to drug-induced stress, characterized by reduced reliance on glycolysis and increased dependence on OXPHOS and fatty acid oxidation for energy production (76, 77). This metabolic shift necessitates corresponding structural and functional adaptations in the mitochondrial network to meet increased energy demands. Specifically, drug-resistant cells enhance their mitochondrial infrastructure through increased biogenesis and functional optimization, thereby supporting the efficiency of both OXPHOS and fatty acid oxidation processes (78). Furthermore, cisplatin-resistant cells typically persist in environments characterized by elevated levels of ROS (79). ROS are oxidizing molecules generated during cellular metabolism. Under normal physiological conditions, they function as signaling molecules; however, when produced in excess, ROS induce oxidative stress and cause cellular damage (80). To adapt to high ROS conditions, drug-resistant cells trigger mitochondrial fusion, resulting in enlarged mitochondria. This morphological adaptation enhances the mitochondrial antioxidant defense capacity, thereby protecting cells from oxidative damage (81). Furthermore, cisplatin-resistant cells activate additional antioxidant pathways, such as through the upregulation of peroxisome proliferator-activated receptor gamma coactivator 1-alpha (PGC-1α), to bolster their antioxidant defense systems. Together, this antioxidant response and mitochondrial fusion create a cooperative resistance cycle that not only counteracts drug toxicity but also sustains cell viability (80, 82) (**Figure 3**). From an immunotherapy perspective, enhanced OXPHOS contributes to EMT, which in turn upregulates programmed death-ligand 1 (PD-L1) expression levels (78). The upregulation of PD-L1 sensitizes otherwise immunotherapy-resistant tumor cells to immune treatment, thereby enhancing its efficacy. However, it is important to note that this beneficial effect of OXPHOS enhancement is accompanied by certain negative consequences, as it simultaneously equips tumor cells with enhanced evasion mechanisms that effectively bypass the cytotoxic effects of chemotherapeutic agents such as cisplatin (78, 83). Thus, while the efficacy of immunotherapy is enhanced, the effectiveness of chemotherapeutic agents may be significantly compromised, presenting a novel therapeutic challenge in oncology. In summary, mitochondrial dynamics play a pivotal role in cell survival by maintaining metabolic homeostasis through fine-tuned regulation of processes such as OXPHOS efficiency and ROS balance, ultimately exerting profound effects on cellular viability (84, 85). The imbalance in mitochondrial dynamics—characterized by enhanced fusion and suppressed fission—is closely associated with cellular metabolic reprogramming during the development of cisplatin resistance. This metabolic reprogramming manifests as increased cellular dependence on OXPHOS and enhanced lipid metabolism. Collectively, these adaptations enable cells to counteract cisplatin’s cytotoxic effects, thereby promoting survival during chemotherapy (86). Therefore, a thorough investigation of mitochondrial dynamics and their interplay with metabolic reprogramming is of critical importance for elucidating the mechanisms of cisplatin resistance and developing novel therapeutic strategies.

## 4 The crosstalk between immunity and mitochondrial dynamics in drug resistance

### 4.1 The tumor immune microenvironment and cisplatin resistance

Cisplatin is a cornerstone chemotherapeutic agent whose efficacy is significantly limited by the development of drug resistance. It is well established that resistance arises not only from cell-intrinsic mechanisms but also through an orchestrated collaboration with the tumor immune microenvironment (TIME).The TIME constitutes a dynamic network composed of immune cells, stromal cells, soluble factors, and the extracellular matrix. Through bidirectional crosstalk with tumor cells, it co-fosters a protective niche that favors therapeutic failure (87). A key underlying mechanism involves tumor-associated macrophages (TAMs). Under the pressure of cisplatin, these cells polarize towards an M2-like, pro-tumoral phenotype. These M2-like TAMs secrete factors such as interleukin-6 (IL-6), which in turn activate anti-apoptotic pathways, including STAT3, within tumor cells. This cascade ultimately blunts cisplatin-induced cell death (88). Of particular importance is the finding that TAM-derived cathepsin B can directly inactivate cisplatin in the extracellular compartment, thereby effectively diminishing the drug’s availability and subsequent intracellular accumulation (89). Furthermore, myeloid-derived suppressor cells (MDSCs) represent another pivotal player. They upregulate the expression of immunosuppressive products such as arginase-1 (ARG-1), inducible nitric oxide synthase (iNOS), and ROS. This activity suppresses the function of cytotoxic T cells and depletes CD8+ T cells, thereby compromising the immune system’s capacity to clear drug-damaged tumor cells (90). Furthermore, cancer-associated fibroblasts (CAFs) contribute to resistance by remodeling the extracellular matrix (ECM) to create a physical barrier that impedes drug penetration, and by delivering pro-survival molecules such as miR-21 via exosomes to directly regulate apoptotic pathways within tumor cells (91). Moreover, this resistant microenvironment is further consolidated by the cisplatin-induced upregulation of PD-L1 and the immunosuppressive activity of regulatory T cells (Tregs) (92). In summary, current research unequivocally positions the TIME as a central driver of cisplatin resistance. The intricate crosstalk between these immune cells and tumor cells ultimately leads to downstream effects that collectively converge to reprogram the core fate-determining processes of tumor cells: namely, cellular metabolism and survival signaling. Notably, the research frontier is beginning to reveal that this microenvironment-mediated resistance is, in large part, executed through the regulation of tumor cell mitochondrial function (93, 94). The secretory and interactive signals from immune cells converge to rewire the mitochondrial biology, metabolism, and death susceptibility of cancer cells, constituting the ultimate defense against cisplatin. This convergence thereby unveils a mitochondria-centric axis for both understanding and therapeutically overcoming this resilience.

### 4.2 Cisplatin resistance orchestrated by the tumor immune microenvironment and mitochondrial dynamics

The function of immune cells within the tumor microenvironment is often regulated by their own mitochondrial dynamics, which in turn indirectly modulates the therapeutic efficacy of cisplatin. Under the hypoxic and acidic conditions of the tumor, both tumor-infiltrating lymphocytes (TILs) and natural killer (NK) cells frequently exhibit excessive mitochondrial fission, thereby promoting their functional exhaustion. PD-1 signaling can suppress this fission process, aiding T cells in maintaining metabolic fitness (6). This reveals that an imbalance in mitochondrial dynamics can impair the tumor-clearing capacity of immune cells, thereby indirectly sheltering cancer cells from chemotherapeutic attack by agents such as cisplatin. Similarly, related studies indicate that bone marrow-derived mesenchymal stem cells (MSCs) can promote chemoresistance in T-cell acute lymphoblastic leukemia (T-ALL) cells by influencing the extracellular signal-regulated kinase (ERK) pathway. This leads to the activating phosphorylation of Drp1 at serine 616 (S616), which in turn drives mitochondrial fission and enhances the drug tolerance of the cancer cells (95). Research in colorectal cancer has revealed that high-mobility group box 1 (HMGB1), engaging its receptor RAGE, activates the ERK1/2 pathway to phosphorylate and activate Drp1, thereby fostering chemoresistance. This pathway is further supported by the association between the RAGE-G82S polymorphism and a hyperphosphorylated state of Drp1 at Ser616 in the tumor microenvironment, highlighting the relevance of Drp1 phosphorylation and immune interactions in mediating cisplatin resistance (96). In hepatocellular carcinoma, Drp1-mediated mitochondrial fission triggers mitochondrial DNA (mtDNA) stress, which promotes the recruitment and polarization of tumor-associated macrophages (TAMs). Furthermore, high Drp1 expression in tumor cells shows a significant positive correlation with TAM infiltration in HCC tissues (97). Collectively, these results not only identify mitochondrial fission as a key driver of TAM infiltration but also imply a potential mechanism by which immuno-metabolic crosstalk, governed by mitochondrial dynamics, contributes to chemoresistance against agents like cisplatin.

## 5 Therapeutic strategies targeting mitochondrial dynamics

### 5.1 Modulating the balance of mitochondrial fusion and fission

The processes of mitochondrial fusion and fission play a dual role in the development of cisplatin resistance. An imbalance in their function is considered a key mechanism underlying this resistance (32, 35, 39). On one hand, restoring cancer cell sensitivity to cisplatin can be achieved by inhibiting the expression of mitochondrial fusion proteins or promoting the expression of fission proteins. For instance, in head and neck squamous cell carcinoma (HNSCC), hypoxia-inducible factor 1α (HIF-1α) binds to the MFF and upregulates its expression. This enhanced fission process, in turn, increases the cells’ sensitivity to cisplatin. Conversely, MFF gene silencing inhibits hypoxia-induced mitochondrial fission and reduces cancer cell sensitivity to cisplatin (98). In ovarian cancer, Piceatannol enhances sensitivity to cisplatin treatment by promoting mitochondrial fission and apoptosis. This effect is achieved through the dephosphorylation of DRP1 at serine residue 637 (99). Collectively, these studies demonstrate that enhancing the expression of specific fission proteins can increase cancer cell sensitivity to cisplatin. Conversely, other evidence indicates that inhibiting the expression of certain fission proteins may also promote cisplatin sensitivity, highlighting the context-dependent nature of these mechanisms. Research demonstrates that combining the DRP1 inhibitor mdivi-1 with cisplatin significantly enhances apoptosis in cisplatin-resistant, end-stage ovarian cancer cells (100). Similarly, in metastatic breast cancer cells, inhibiting the activity of the mitochondrial fission protein DRP1 and nuclear factor erythroid 2-related factor 2 (Nrf2) restored sensitivity to cisplatin and effectively suppressed the metastatic awakening process (40). In summary, targeted regulation of mitochondrial dynamics—the balance of fusion and fission—effectively increases sensitivity to cisplatin. This strategy hinges on a deeper understanding of the mitochondrial role in apoptosis and how morphological shifts govern drug resistance. Furthermore, developing small-molecule agonists or inhibitors (e.g., mdivi-1) that target specific proteins involved in mitochondrial fusion or fission offers a precise method to modulate mitochondrial morphology. These compounds represent promising adjuvant agents that could enhance cisplatin efficacy, providing novel strategies for cancer therapy.

### 5.2 Targeting mitophagy

The functional synergy between mitophagy and mitochondrial dynamics collectively influences cisplatin resistance, highlighting the importance of targeting mitophagy. Research has found that upregulating ceramide synthase 6 (CerS6) expression markedly suppresses mitophagy and promotes mitochondrial fission in oral squamous cell carcinoma, thereby inducing apoptosis in resistant cells (101). Similarly, treatment with the mitochondria-targeting aggregation-induced emission molecule DP-PPh_3_ effectively blocks autophagic flux by preventing autophagosome degradation, thereby overcoming cisplatin resistance in non-small cell lung cancer cells (102). Similarly, in liver cancer, mitophagy inhibitors also enhance sensitivity to cisplatin and induce tumor cell apoptosis (103, 104). Collectively, these studies demonstrate that inhibiting mitophagy can enhance tumor cell sensitivity to cisplatin. Conversely, and somewhat paradoxically, promoting mitophagy can also induce apoptosis in resistant cells. Studies have revealed that mitochondria-targeted platinum(II) complexes can activate mitophagy and trigger apoptosis mediated by endoplasmic reticulum stress in cisplatin-resistant cells (105). Therefore, specifically targeting the process of mitophagy can effectively induce apoptosis in cisplatin-resistant cells. This mechanism highlights the critical role of mitophagy in regulating cell death and offers a novel therapeutic strategy to overcome cisplatin resistance.

### 5.3 Targeting mitochondrial metabolism and energy production

Given the significant role of the interplay between mitochondrial dynamics and cellular energy metabolism in mediating cisplatin resistance, and considering that resistant cells typically rely on OXPHOS rather than glycolysis, targeting OXPHOS or impairing metabolic flexibility have emerged as pivotal strategies to enhance cisplatin sensitivity (106, 107). In cisplatin-resistant lung cancer cells, OXPHOS activity is significantly elevated. Treatment with the hexokinase (HK) inhibitor 2-deoxyglucose (2-DG) effectively suppresses OXPHOS levels in these cells, thereby enhancing their sensitivity to cisplatin (108). In ovarian cancer cells, inhibiting their ability to switch between OXPHOS and glycolysis enhances the efficacy of platinum-based chemotherapy (109). Collectively, these findings demonstrate that suppressing OXPHOS in resistant cells enhances their sensitivity to cisplatin. Similarly, reducing ATP levels in these cells produces a comparable effect. Research indicates that metformin inhibits the p32/OPA1 axis, thereby reducing ATP synthesis and inducing mitochondrial fission. When used in combination with cisplatin, metformin significantly diminishes the viability of resistant cells (32). Therefore, targeted intervention in mitochondrial metabolism and energy production can initiate a cascade of events related to mitochondrial dynamics. These changes not only enhance mitochondrial function but also increase cellular sensitivity to cisplatin, ultimately improving the drug’s therapeutic efficacy against tumors. This interventional strategy provides a novel approach for optimizing mitochondrial function and opens new avenues for the clinical application of chemotherapeutic agents like cisplatin, showing significant potential for future cancer therapy.

### 5.4 Mitochondria-targeted drug delivery systems

The development of mitochondria-targeted nanoparticles through nanocarrier systems can circumvent conventional drug resistance mechanisms. Specifically, cisplatin-loaded nanoparticles (NPcis) exploit endocytic pathways for drug delivery, bypassing LRRC8A-mediated resistance and effectively eliminating resistant cells (110). Similarly, when a Pt(IV) prodrug—a type of platinum complex—is conjugated with the mitochondria-targeting ligand triphenylphosphine and specific peptide molecules, the resulting compound effectively disrupts mitochondrial metabolism. This disruption subsequently activates intrinsic apoptotic pathways, ultimately inducing cell death. This multi-level mechanism of action enhances the precision of drug targeting to diseased cells and thereby offers novel therapeutic avenues for cancer treatment (111, 112). Furthermore, in photodynamic therapy (PDT), the application of specific mitochondria-targeted photosensitizers can efficiently generate ROS upon irradiation at specific wavelengths. These ROS precisely target and damage mitochondria within tumor cells, thereby significantly disrupting cellular energy metabolism and ultimately reducing cell viability. This precisely targeted mitochondrial damage not only directly compromises tumor cell viability but also significantly potentiates the efficacy of platinum-based chemotherapy, enabling more effective tumor suppression and ultimately improving overall therapeutic outcomes (113, 114). Consequently, the innovative approach of utilizing nanoparticle delivery systems to overcome cisplatin resistance demonstrates high potential for clinical translation.

### 5.5 Preclinical *in vivo* models and clinical translation of therapeutic targeting of mitochondrial dynamics

Preclinical *in vivo* and clinical translational studies form a critical translational bridge, validating the therapeutic promise of targeting mitochondrial dynamics to overcome cisplatin resistance. Studies in xenograft mouse models demonstrated the critical role of mitochondrial dynamics in cisplatin resistance. In L1210/DDP leukemia models, the Drp1 inhibitor Mdivi-1 attenuated cisplatin-induced cell death, caspase-3 activation, and ROS elevation, whereas promoting mitochondrial fission reversed these effects (31). Similarly, in SKOV3/DDP ovarian cancer models, specific knockout of the fusion protein Mfn2 restored tumor sensitivity to cisplatin (38). These *in vivo* findings collectively elucidate the pivotal role of targeting mitochondrial dynamics proteins in reversing cisplatin resistance and underscore their therapeutic potential. Metformin is extensively utilized in clinical practice as a first-line therapy for glycemic control (115). Furthermore, numerous studies indicate its efficacy in counteracting cisplatin resistance, suggesting promise for clinical application (116, 117). In studies using A549/DDP xenograft models, metformin was found to induce mitochondrial fission and deplete ATP, thereby sensitizing the tumors to cisplatin and leading to significant tumor growth inhibition upon combination treatment (32). Phase II clinical trial results demonstrated a modest improvement in overall survival with the metformin-cisplatin combination in non-small cell lung cancer patients, and shown a favorable tolerability profile in head and neck squamous cell carcinoma (116, 118).These findings underscore the significant translational value of targeting mitochondrial dynamics in cancer therapy.

In summary, while most research on targeting mitochondrial dynamics to overcome cisplatin resistance remains preclinical, the field is rapidly evolving beyond the sole targeting of dynamics proteins to encompass closely linked processes like mitophagy and mitochondrial metabolism. Furthermore, the continuous advancement of nanocarrier technology is poised to refine these therapeutic strategies. This review consolidates current pharmacological strategies that target mitochondrial dynamics to combat cisplatin resistance (**Table 2**). By integrating both preclinical and clinical data, it affirms the synergistic therapeutic promise of combining cisplatin with mitochondrial dynamics-targeted agents (**Table 3**).

**Table 2:** Advances in drug development targeting mitochondrial dynamics.

Pharmacologic agent/intervention	Target/mechanism	Cancer model	Main effect	Development phase	References
Piceatannol	Activate the p53-NOXA pathway, degrade XIXP, Stimulate mitochondrial fission	Ovarian cancer	sensitize to cisplatin, reverse drug resistance, induce apoptosis	Preclinical research	(99)
Mdivi-1	The Drp1-independent dual mechanism: 1. inhibits DNA replication, exacerbates cisplatin-induced replication stress 2. inhibits mitochondrial respiration, triggers mitochondrial uncouplingand and induces mitochondrial swelling	Ovarian cancer	synergistically upregulates Noxa, induces physical mitochondrial swelling and induces apoptosis to overcome platinum-based and multi-drug resistance	Preclinical research	(100)
INF2-targeting compound	Inhibits ubiquitination-mediated degradation	Cholangiocarcinoma (CCA)	inhibits mitochondrial hyperfusion	Lead compound optimization	(35)
Metformin	Inhibits the p32/OPA1 signaling axis	Non-small cell lung cancer (NSCLC)	reduces ATP levels, reverses drug resistance, induces apoptosis	Phase II Clinical Trial (NCT03874000)	(32)
Autophagy inhibitor (CQ)	Inhibits the BCAT1-Leu-mTOR-autophagy signaling axis	Cervical cancer/ Liver cancer	Inhibits autophagy, reverses drug resistance, induces apoptosis	Phase II Clinical Trial (NCT04333914)	(104)
DP-PPh_3_	Induces mitochondrial dysfunction、blocks autophagic flux	Non-small cell lung cancer (NSCLC)	potently and selectively kills cisplatin-resistant cells with low *in vivo* toxicity	Preclinical research	(102)

**Table 3:** Advances in cisplatin combination with mitochondrial dynamics-targeted agents.

Cisplatin-based combination therapies	Cancer model	Main effect	Development phase	References
Mdivi-1	Kidney cancer	Synergizes with cisplatin to induce tumor cell apoptosis	Preclinical research	(119)
ABT - 199	Oral tongue squamous cell carcinoma	Combination with cisplatin enhances the induction of tumor cell apoptosis compared to either agent alone	Preclinical research	(120)
Piceatannol	hepatocellular cancer/Ovarian cancer	Synergizes with cisplatin to induce tumor cell apoptosis	Preclinical research	(99, 121)
Metformin	head and neck squamous cell carcinoma(HNSCC)	Patients exhibited good tolerance to the cisplatin-based combination therapy	Phase II Clinical Trial (NCT02949700)	(116)

## 6 Conclusions and perspectives

Remodeling of mitochondrial dynamics has emerged as a pivotal hub in cisplatin resistance. An imbalance between fusion and fission not only promotes the formation of hyperfused networks but, conversely, excessive fission can also facilitate the development of drug resistance. This review extends beyond examining the cisplatin resistance mechanisms directly driven by imbalances in mitochondrial dynamics to incorporate their interplay with mitophagy, metabolism, and the tumor immune microenvironment, thereby synthesizing their synergistic mechanisms from a broader perspective. Furthermore, it provides a systematic discussion on therapeutic strategies, including modulating mitochondrial fusion/fission, targeting mitophagy, intervening in mitochondrial metabolism and energy supply, and developing mitochondrial-targeted drug delivery systems. By synthesizing clinically relevant data, this work underscores the significant translational value of developing drugs targeting mitochondrial dynamics. Nonetheless, numerous promising avenues for future investigation remain. For instance, developing dynamic monitoring techniques using novel fluorescent probes (e.g., DHX-Fe) to track real-time morphology-metabolism changes in live cells would help elucidate the spatiotemporal dynamics of drug resistance development (122); Future studies should focus on constructing organelle interaction networks to investigate the role of endoplasmic reticulum-mitochondria contact sites (MAMs) in cisplatin resistance development, and to examine the regulatory mechanisms of the INF2-ER-mitochondria axis (123). Undoubtedly, these research directions provide novel avenues for overcoming cisplatin resistance.

However, translating mitochondrial dynamics-targeted strategies into clinical treatments for reversing cisplatin resistance faces substantial challenges. The specific mechanisms by which mitochondrial dynamics drive resistance vary significantly across cancer types and even within individual tumors. Moreover, precisely modulating the balance between fission and fusion remains difficult. These challenges underscore the critical need to develop biomarker-based stratification strategies. Consequently, a primary objective in drug development is to learn how to selectively modulate mitochondrial function in diseased cells without affecting healthy ones.

This review integrates recent advances in mitochondrial dynamics to build a theoretical and translational framework for overcoming cisplatin resistance. Future research should focus on three key areas: dynamic monitoring, targeted delivery, and clinical validation, to establish novel tumor treatment strategies based on the precise targeting of mitochondria.
